# Evaluation of the relationship between vitamin D levels with oocyte quality in breast cancer women: a cross-sectional study

**DOI:** 10.1038/s41598-023-39341-w

**Published:** 2023-07-26

**Authors:** Mahshid Gharagozloo, Shahideh Jahanian Sadatmahalleh, Mehri Kalhor, Firouzeh Ghaffari, Fatemeh Hasani, Nadia Jahangiri, Malihe Nasiri, Ahmad Khosravi

**Affiliations:** 1 Department of Reproductive Health and Midwifery, Faculty of Medical Sciences, Tarbiat Modares University, Tehran, 14115-111 Iran; 2 Department of Midwifery, School of Nursing and Midwifery, Shahid Beheshti University of Medical Sciences, Tehran, Iran; 3 Department of Endocrinology and Female Infertility, Reproductive Biomedicine Research Center, Royan Institute for Reproductive Biomedicine, ACECR,, Royan Allay, Eastern Hafez St., Banihashem Sq., Resalat Highway, Tehran, 1665659711 Iran; 4 Department of Embryology, Reproductive Biomedicine Research Center, Royan Institute for Reproductive Biomedicine, ACECR, Tehran, Iran; 5 Department of Basic Sciences, Faculty of Nursing and Midwifery, Shahid Beheshti University of Medical Sciences, Tehran, Iran; 6 Department of Epidemiology, School of Public Health, Ophthalmic Epidemiology Research Center, Shahroud University of Medical Sciences, Shahroud, Iran

**Keywords:** Health care, Medical research, Oncology, Risk factors

## Abstract

Recent evidence suggests that vitamin D deficiency could play an important role in the development of non-skeletal diseases, including cancer. Vitamin D also affects the function of the reproductive system. In the present study, the relationship between 25(OH)D levels with oocyte quality in Breast Cancer (BC) women and control group have been investigated. After initial evaluations, ovarian stimulation began with the GnRH antagonist protocol in the BC group (N = 16) and control group (N = 16). The serum and follicular fluid (FF) 25(OH)D levels were measured at the time of oocyte retrieval and their relationship to oocyte quality was examined. The mean levels of serum and FF 25(OH)D in BC women were significantly lower than in the control group (22.26 ± 7.98 vs. 29.61 ± 9.12, P = 0.02, 21.65 ± 7.59 vs. 28.00 ± 9.05, P = 0.04, respectively). There was a significant correlation between the levels of 25(OH)D in FF and serum in BC women (r = 0.873, P < 0.001). But there was no correlation between the serum or FF 25(OH)D levels with the parameters related to oocytes (P > 0.05). In the BC women, the number of dysmorph and highly dysmorph oocytes was higher than in the control group (P < 0.001). Women with BC referring to infertility centers for fertility preservation are more likely to be deficient in serum 25(OH)D level; this subsequently affects the FF 25(OH)D level. However, serum and FF 25(OH)D levels may not be suitable indicators for examining maturity and quality of oocytes in terms of morphology in BC women, and the poor morphological quality of oocytes in BC women may be due to other factors.

## Introduction

Breast cancer (BC) is the most common cancer diagnosed among women in the world^1^. Cancer mortality has declined due to early detection and therapeutic advances in recent decades^2^. More survivors after completing oncotherapy face the establishment of a family due to increased cancer survival and delayed childbearing^3^. It is worth noting that cancer treatments, including pelvic radiation, after chemotherapy and surgery can cause iatrogenic damage to the reproductive system, and so, pose a threat to female fertility^4^. The American Society of Clinical Oncology recommends that oncologists discuss fertility preservation options with their patients as early as possible before treatment starts^5^.

Studies show that BC patients have significantly lower serum levels of 25-OH vitamin D than healthy subjects. Low serum 25-OH vitamin D levels are associated with aggressive breast cancer phenotypes^6^. So that an inverse relationship was found between the serum level of 25(OH) D and the risk of breast cancer. Also, a statistical difference was observed between different vitamin D receptors (VDRs) genotypes and circulating levels of 25(OH)D among women with breast cancer^7^.

Vitamin D is a steroid molecule that regulates the expression of a large number of genes by interacting with vitamin D receptors (VDRs)^8^. VDRs are found in most tissues and cells in the body. The main role of vitamin D is to regulate bone metabolism and homeostasis of calcium and phosphorus^9^. Recent evidence suggests that vitamin D can also have non-skeletal actions, including an important role in cancers^10^. Vitamin D has multiple anticarcinogenic roles in BC that are well-described at the molecular level^11^.

The interaction between vitamin D and reproduction is based on the presence of both VDR and 1α-hydroxylase (CYP27B1) in various tissues of the reproductive system in both sexes. So that presence of VDRs and 1α-hydroxylase (CYP27B1) in various tissues of the male and female reproductive organs indicates the role of vitamin D in the function of the reproductive system. CYPR1 in reproductive organ is involved in vitamin D activation, as its expression progressively decreases in association with the reproductive organ damage^12^. VDRs are present in the glandular epithelial cells of endometrium, fallopian epithelial cells, granulosa cells and cumulus oophorus cells of the ovary^8,12^. Vitamin D levels of follicular fluid (FF), which serves as the biochemical microenvironment of the oocyte before ovulation, correlate with the quality of the oocyte and oocyte competence^13^.

The studies show that with presence of VDR in female reproductive tissue, vitamin D is an important role in female reproduction. So that vitamin D deficiency may change AMH expression and its serum level^14^. On the other hand, vitamin D plays a role in inducing apoptosis, stimulating cell differentiation, anti-inflammatory and anti-proliferative effects, and inhibiting angiogenesis, invasion and metastasis in cancer^15^. Therefore, vitamin D deficiency in women with breast cancer can affect their fertility and the quality of their ovaries^16^.

Considering the anticarcinogenic roles of vitamin D in BC and its role in the function of the female reproductive system and taking into account that in various physiological and pathological conditions, the metabolism and signaling of vitamin D are different^17^, the present study was conducted to evaluate the possible relationship between vitamin D levels with oocyte quality in BC women comparing to the control group.

## Results

The characteristics of both groups of women are given in Table 1. As shown, there are no significant differences in demographic information, fertility, hormone profile, ovarian stimulation drugs, physical activity and sun exposure between the groups (P > 0.05).

**Table 1:** Comparison of the participants’ characteristics between the breast cancer and control groups.

Variables	Control group (N = 16)	Breast cancer group (N = 16)	P-value
	Mean ± SD or n (%)	Mean ± SD or n (%)	
	29.43 ± 4.67	30.43 ± 3.18	0.48*
Age (years)	23.41 ± 2.13	23.21 ± 2.13	0.62**
BMI (kg/m^2^)			0.28***
Occupation	6 (37.5)	9 (56.3)	
Unemployed	10 (62.5)	7 (43.7)	
Employed			0.34***
Education	1 (6.3)	3 (18.8)	
Diploma	1 (6.3)	2 (12.5)	
Associate	9 (56.3)	7 (43.8)	
Bachelor	4 (25.0)	3 (18.8)	
Master	1 (6.3)	1 (6.3)	
Doctorate			0.36***
Residence	12 (75.0)	14 (87.5)	
Tehran	4 (25.0)	2 (12.5)	
Another city			0.89***
Season	4 (25.0)	5 (31.3)	
Spring	6 (37.5)	7 (43.8)	
Summer	3 (18.8)	2 (12.5)	
Autumn	3 (18.8)	2 (12.5)	
Winter	12.62 ± 1.20	12.56 ± 1.15	0.82**
Menarche (years)	6.75 ± 1.18	6.56 ± 1.31	0.63**
Menstruation duration (days)	28.93 ± 1.87	28.75 ± 2.59	0.90**
Menstruation interval (days)	1.63 ± 4.24	2.52 ± 4.46	0.79*
LH (mIU/mL)	1.92 ± 6.16	1.20 ± 5.16	0.19*
FSH (mIU/mL)	2.48 ± 0.80	3.03 ± 1.22	0.14*
AMH (ng/mL)	4.62 ± 1.36	4.62 ± 1.20	1.00*
Antagonist (days)	1692.18 ± 776.51	1964.06 ± 817.56	0.40**
Gonadotropin (75 IU)	157.03 ± 28.12	168.75 ± 33.54	0.24**
Starting dose of gonadotropin (IU)	9.25 ± 1.91	9.50 ± 1.67	0.69*
Duration of ovarian stimulation (days)			0.38***
Physical activity (fast walking, 20 min a day)	3 (18.8)	1 (6.3)	
Everyday	3 (18.8)	3 (18.8)	
Twice a week	1 (6.3)	2 (12.5)	
Four times a week	5 (31.3)	7 (43.8)	
Irregular and sometimes	4 (25.0)	3 (18.8)	
None			0.61***
Duration of exposure to sunlight	2 (12.5)	4 (25.0)	
Not exposed	11 (68.8)	8 (50.0)	
10 min to 1 h	3 (18.8)	2 (12.5)	
1 to 2 h	0 (0)	2 (12.5)	
More than 2 h			0.73***
Sun exposure time	3 (18.8)	3 (18.8)	
7–10 am	7 (43.8)	5 (31.3)	
10 am–15 pm	6 (37.5)	8 (50.0)	
15–17 pm			0.60***
Part of the body exposed to sunlight	2 (12.5)	4 (25.0)	
Face	1 (6.3)	0 (0.0)	
Wrist	13 (81.3)	12 (75.0)	
Face and wrist			0.75***
Use of sunscreen	5 (31.3)	4 (25.0)	
No	6 (37.5)	7 (43.8)	
Sometimes	3 (18.8)	2 (12.5)	
Mostly	2 (12.5)	3 (18.8)	
Always			

Significant at the 0.05 level (two-tailed).

*n* number, *%* percentage, *SD* standard deviation, *BMI* body mass index, *LH* luteinizing hormone, *FSH* follicle-stimulating hormone, *AMH* anti-mullerian hormone.

*T-test, **Mann–Whitney, ***Chi-squared.

As displayed in Table 2, the mean levels of serum and FF 25(OH) D in the BC group are significantly lower than in the control group (22.26 ± 7.98 vs. 29.61 ± 9.12 and 21.65 ± 7.59 vs. 28.00 ± 9.05, P > 0.05, respectively). The Pearson's correlation analysis results, showed a significant correlation between the levels of 25(OH) D in serum and FF in BC women (correlation coefficient r = 0.873, P < 0.001) and Control group (correlation coefficient r = 0.886, P < 0.001).

**Table 2:** Comparison of serum and FF 25(OH) D levels and oocyte parameters between the two groups.

Variables	Control group (N = 16)	Breast cancer group (N = 16)	P-value
	Mean ± SD or n (%)	Mean ± SD or n (%)	
Serum 25(OH) D level	29.61 ± 9.12	22.26 ± 7.98	0.02*
FF 25(OH)D level	28.00 ± 9.05	21.65 ± 7.59	0.04*
Number of follicles > 16 mm	4.12 ± 2.27	4.68 ± 2.60	0.52**
Number of oocytes retrieved	12.68 ± 6.37	13.43 ± 8.28	0.77**
Number of MII oocytes	10.56 ± 5.42	9.87 ± 5.78	0.73**
Oocyte maturity	83.81 ± 9.85	76.70 ± 16.60	0.15*
Morphology of MII oocytes			< 0.001***
Morph	29 (17.2)	10 (6.5)	
Slightly dysmorph	98 (58.0)	65 (41.9)	
Dysmorph	40 (23.7)	43.2 (67)	
Highly dysmorph	2 (1.2)	13 (8.4)	

Significant at the 0.05 level (two-tailed).

*n* number, *%* percentage, *SD* standard deviation, *FF* follicular fluid, *25(OH)D* 25-hydroxyvitamin D, *MII* metaphase II.

*T-test, **Mann–Whitney, ***Chi-squared.

There was no significant difference in terms of the number of follicles > 16 mm, number of oocytes retrieved, MII oocytes and oocyte maturity between the two groups. Although the number of morph and slightly dysmorph oocytes in the BC group was significantly less than in the control group, the number of dysmorph and highly dysmorph oocytes in these women was statistically higher than in their control counterparts (P < 0.001) (Table 2).

In the assessment of correlation between variables (Table 3), in BC women, as in the control group, no significant correlation was observed between the serum and FF 25(OH)D levels with the number of follicles > 16 mm, number of oocytes retrieved, number of MII oocytes, oocyte maturity and morphology of MII oocytes (P > 0.05).

**Table 3:** Correlation of serum and FF 25(OH) D levels with oocyte parameters in the two groups.

Variables	Control group (N = 16)		Breast cancer group (N = 16)	
	Correlation coefficient (r)	P-value	Correlation coefficient (r)	P-value
Serum 25(OH) D level and oocyte parameters				
Number of follicles > 16 mm	−0.154	0.57*	0.436	0.09*
Number of oocytes retrieved	0.046	0.86*	0.438	0.09*
Number of MII oocytes	0.173	0.52*	0.359	0.17*
Oocyte maturity	0.450	0.08*	−0.032	0.90*
Morphology of MII oocytes				
Morph	−0.161	0.55**	−0.217	0.43**
Slightly dysmorph	−0.021	0.93**	0.123	0.42**
Dysmorph	0.122	0.65**	0.215	0.64**
Highly dysmorph	0.252	0.34**	−0.364	0.16**
Follicular fluid 25(OH) D level and oocyte parameters				
Number of follicles > 16 mm	−0.274	0.30*	0.393	0.13*
Number of oocytes retrieved	−0.132	0.62*	0.446	0.08*
Number of MII oocytes	−0.018	0.94*	0.268	0.31*
Oocyte maturity	0.349	0.18*	−0.180	0.50*
Morphology of MII oocytes				
Morph	−0.253	0.34**	0.062	0.82**
Slightly dysmorph	−0.097	0.72**	−0.013	0.96**
Dysmorph	0.089	0.74**	−0.231	0.38**
Highly dysmorph	0.028	0.91**	−0.361	0.16**

Significant at the 0.05 level (two-tailed).

*MII* metaphase II, *n* number, *FF* follicular fluid, *25(OH)D* 25hydroxyvitamin D.

*Pearson, **Spearman.

Moreover, there were statistically significant differences in the mean level of serum 25(OH)D between the morph, slightly dysmorph, dysmorph and highly dysmorph oocytes in both groups (P < 0.001). In the BC women, Bonferroni post-hoc comparisons showed that the mean level of serum 25(OH) D in the highly dysmorph oocytes was significantly lower than in the morph oocytes (13.87 ± 0.00 vs. 21.10 ± 0.00, P = 0.01), slightly dysmorph oocytes (13.87 ± 0.00 vs. 24.46 ± 6.60, P < 0.001) and dysmorph oocytes (13.87 ± 0.00 vs. 25.24 ± 9.06, P < 0.001). Also the mean level of serum 25(OH) D in the dysmorph oocytes was significantly higher than slightly dysmorph oocytes (25.24 ± 9.06 vs. 24.46 ± 6.60, P = 0.02). In the control group, the mean level of serum 25(OH) D in the morph oocytes was significantly less than in the dysmorph oocytes (25.38 ± 4.15 vs. 30.12 ± 3.47, P = 0.03) (Table 4).

**Table 4:** Comparison of the morphology of MII oocytes in terms of serum Vitamin D level in the two groups.

Group	Morphology of MII oocytes	Serum vitamin D level, mean ± SD	P-value*	Pairwise comparison, P-value**
Breast cancer	Morph	21.10 ± 0.00	< 0.001	M vs. SD: 0.46 M vs. D: 1.00 M vs. HD: 0.01 D vs. SD: 0.02 D vs. HD: < 0.001 SD vs. HD: < 0.001
	Slightly dysmorph	24.46 ± 6.60		
	Dysmorph	25.24 ± 9.06		
	Highly dysmorph	13.87 ± 0.00		
	Total	21.16 ± 3.91		
Control	Morph	25.38 ± 4.15	0.02	M vs. SD: 0.26 M vs. D: 0.03 M vs. HD: 0.28 D vs. SD: 1.00 D vs. HD: 1.00 SD vs. HD: 0.91
	Slightly dysmorph	29.04 ± 11.38		
	Dysmorph	30.12 ± 3.47		
	Highly dysmorph	35.20 ± 0.00		
	Total	29.61 ± 9.12		

Significant at the 0.05 level (two-tailed).

*MII* metaphase II, *M* morph, *SD* slightly dysmorph, *D* dysmorph, *HD* highly dysmorph.

*Kruskal–Wallis test.

**Kruskal–Wallis test followed by Bonferroni post-hoc test.

## Discussion

Past evidence suggests that vitamin D has a potential role in ovarian follicular development and function from primordial follicle activation to ovulation, and ultimately generating mature and competent oocytes for fertilization^13,17^. In clinical studies, the relationship between vitamin D levels in the circulation and the follicle or oocyte parameters may be significantly different among patients due to various physiological and pathological conditions^17^. The present research results revealed no correlation between the serum and FF 25(OH)D levels with the number of oocytes retrieved, follicles > 16 mm, and MII Oocytes, as well as oocyte maturity and morphology of MII oocytes in both of the study groups.

The results of our study showed that in BC women, as in the control group, the serum and FF 25(OH)D levels were correlated, and this indicates that peripheral vitamin D status is a reliable indicator of intraovarian 25(OH)D. Dehghani Firouzabadi et al. found a significant correlation between the serum and FF 25(OH) D levels (r = 0.83; P = 0.001)^18^. Also Ozkan et al. reported a correlation between the serum and FF 25(OH) D levels (r = 0.94, P < 0.001), demonstrating that FF 25(OH) D levels are actually a reliable reflection of vitamin D reserves in the body^19^. Aleyasin et al.^20^ and Ciepiela et al.^13^ reported similar results.

In this study, the mean level of serum 25(OH) D in the BC women referring to the Royan Institute for fertility preservation was significantly lower than in the control group. According to Voutsadakis’s study, there is an association between vitamin D deficiency and BC at diagnosis and may be linked pathophysiologically with BC development or progression^11^. De La Puente-Yagüe et al. summarized the mechanisms of action of vitamin D in BC as follows: (a) decreases cell proliferation and increases cell maturation and apoptosis, (b) suppresses inflammation and reduces the accumulation of inflammatory cells, (c) inhibits angiogenesis and regulates insulin secretion and action^15^. In the present study, according to the correlation between serum and FF 25(OH) D levels, the mean level of FF 25(OH) D was lower in the BC group comparing to the control group; this difference can affect oocyte competence^13^. However, the existence and importance of vitamin D metabolism and signaling pathway during folliculogenesis and oogenesis, and the mechanisms of vitamin D effect on oocyte competence remain unclear.

The results of Castiglione Morelli et al.'s study showed that FF has a different metabolic composition in cancer patients as compared to healthy controls^21^. Glucose is an essential metabolite for the Cumulus Oocyte Complex (COC), which provides substrates for energy production to the oocyte^22^. The effect of altering levels of glucose metabolism is probably one of the main reasons for diminished oocyte competence and reduced fertility^23^. Anifandis et al. showed a significant negative correlation between FF vitamin D levels with FF glucose levels, probably through affecting insulin action, which resulted in modulation of FF glucose metabolism^23,24^. On the other hand too high or too low glucose concentrations are detrimental to oocyte maturation, granulosa growth and cumulus cells, which can directly affect oocyte competence^24^. Evidence show that vitamin D have a regulatory role in ovulatory dysfunction, insulin resistance and hyperandrogenism. Evidence has shown that calcitriol increases insulin receptor expression, insulin synthesis and secretion, insulin sensitivity, and reduces the production of pro-inflammatory cytokines. Abnormal fluctuations in intracellular calcium levels in insulin target organs may contribute to peripheral insulin resistance, through impaired insulin signal transduction and glucose transporter function^7^. Therefore, it is possible that in women with BC, altered levels of FF 25(OH) D affect FF glucose levels, and thereby, oocyte competence.

The findings of Polyzos et al. indicated that there was no significant difference in the number of oocytes retrieved and the number of MII oocytes among the infertile women with serum vitamin D levels less than 20 ng/ml and more than 20 ng/ml^25^. Also, Ciepiela et al. reported that there was a significant difference in the number of oocytes retrieved, the number of MII, MI oocytes, estradiol, and progesterone on the day of hCG between infertile women with serum 25(OH) D levels less than 20 ng/ml and more than 20 ng/ml^13^. According to Farzadi et al.'s study, no significant correlation was observed between FF 25(OH) D levels with the number of oocytes, the number of fertilized oocytes, duration of infertility, and estradiol levels^26^. Our findings showed that in the BC women, as in the control group, there was no correlation between serum and FF 25(OH) D levels with the number of follicles > 16 mm, the number of oocytes retrieved and oocyte maturity. In contrast, Castiglione Morelli et al. reported a different trend of the correlations between the total number of follicles and oocytes quality with some metabolites in BC patients^21^. This difference is because cancer leads to disturbances in the metabolism of amino acids, lipids, organic acids, and the serum level of 25(OH) D and glucose in the follicular fluid, which affects the egg and its quality. According to the mentioned results, a certain threshold of the amount of vitamin D affects fertility outcomes. So, it seems that more studies are needed in this field. Also, the sample size of the current study was small, and the study researchers recommend that studies with a larger volume be conducted.

In the present study, the relationship between serum and follicular fluid 25(OH) D levels with the quality of oocytes in terms of morphology was investigated in BC women compared to the control group. The results showed that there is no correlation between the serum and FF 25(OH) D levels of women with BC, as in the control group, with the number of morph, slightly dysmorph, dysmorph and highly dysmorph oocytes. Also the mean of serum 25(OH) D levels in the morphology categories of oocytes was investigated separately in each group. Although in the BC women, the mean serum 25(OH) D level in highly dysmorph oocytes was significantly lower than in the other three categories of oocytes, but it should be considered that these oocytes belonged only to one BC woman. The mean serum 25(OH) D level in the slightly dysmorph group was significantly lower than in the dysmorph group (P < 0.05). In the control group, the mean serum 25(OH) D level in the morph group was significantly lower than in the dysmorph group (P < 0.05), and the mean serum 25(OH) D level in the dysmorph and highly dysmorph groups was at the replete level (30.12 ± 3.47 and 35.20 ± 0.00, respectively). Therefore, it cannot be concluded that increasing the serum level of 25(OH) D improves the morphology of oocytes. In the study of Farzadi et al., there was no significant correlation between FF 25(OH) D level and oocytes quality based on morphological criteria in infertile women^26^. But results in the study of Han Jy et al. showed that vitamin D FF levels were significantly higher in women with decreased ovarian reserve. The promoter region of the AMH gene is very similar to vitamin D response elements, and it has been suggested that vitamin D concentration may be related to AMH expression^27^. In the present study, there was no correlation between serum and FF 25(OH) D levels with the morphology of MII oocytes (Table 3). So, serum and FF 25(OH) D levels probably cannot be suitable indicators for examining the morphology of oocytes in BC women. These contradictory findings may be because the optimal level of vitamin D FF in accordance with ovarian reserve, are not considered. Therefore, it is necessary to determine an optimal threshold for vitamin D levels in follicles.

A study by Zaire et al. showed that vitamin D3 and 25-hydroxyvitamin D3 can act as substrates for VD3 production and autocrine role in the regulation of follicle development. As a result, further research using follicular culture on protein production and cellular function of vitamin D biosynthetic protein, in addition to signaling pathways and autocrine function of vitamin D is needed^28^.

Our findings indicated that the quality of oocytes based on morphological criteria in women with BC was significantly different from the control group. In BC women, the number of dysmorph and highly dysmorph oocytes was higher and the number of morph and slightly dysmorph oocytes was lower than in the control group. This can be caused by other factors that affect the morphology of oocytes in BC women. For example, Bercaire LMN et al. reported that letrozole is a risk factor for worse oocyte morphology^29^. Also, the study of Hossein Rashidi et al. showed that in the letrozole group estradiol level was significantly lower and testosterone significantly higher than in the control group. The number of retrieved oocytes, MII oocytes, and high grade oocyte numberwas significantly lower in the letrozole group (p < 0.05)^30^.

This study had several limitations, including measuring 25(OH) D level only from the lead follicles, small sample size, and not measuring Vitamin D Binding Globulin (VDBP) levels. This is a cross-sectional exploratory study and the sample size is small for some factors. The strength of this research was that, for the first time, the level of FF 25(OH) D was investigated in the BC women referring for fertility preservation.

## Conclusion

The research results showed that BC women referring to infertility centers for fertility preservation are more likely to be deficient in serum 25(OH) D; this subsequently affects the FF 25(OH) D level. Therefore, these people need more attention due to the time limit to start their cancer treatment. However, serum and FF 25(OH) D levels may not be suitable indicators for examining the maturity and quality of oocytes in terms of morphology in women with BC, and the poor morphological quality of oocytes in BC women may be due to other causes, which needs further investigations to find clear-cut answers in this regard.

## Methods

This cross-sectional study has been approved at the Research Ethics Committee of Tarbiat Modares University of Medical Sciences, Tehran, Iran (Ethics code: IR.MODARES.REC.1398.157). The study was performed on 32 eligible women from June 2020 to July 2021 at Royan Institute, Tehran, Iran.

**Figure 1:**
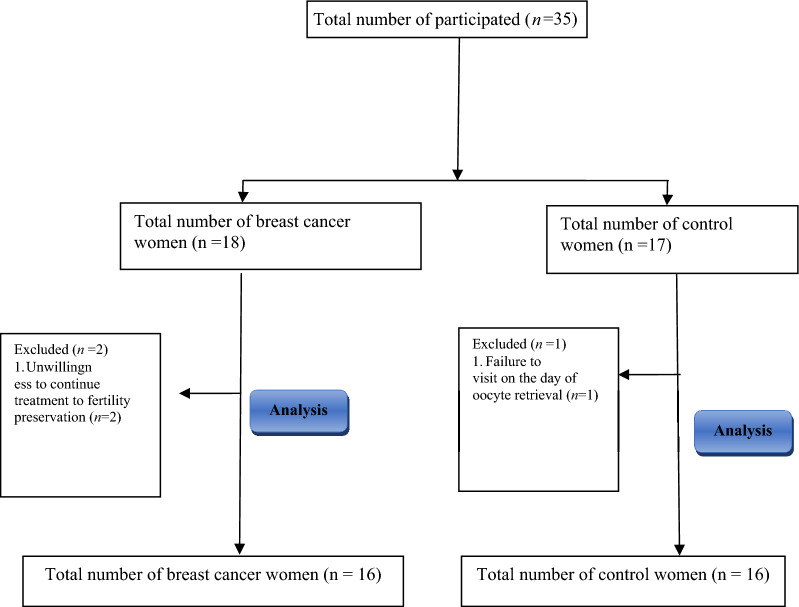
Flow chart of this cross-sectional study.

The participants consisted of two groups:

(1) A group of 16 nulliparous BC women who decided to use oocyte cryopreservation to fertility preservation because they were at risk of losing their fertility due to cancer treatment. Their cancer was diagnosed by histological examination and the stage of the disease was definitive. They had not received any neoadjuvant chemotherapy or radiation therapy.

(2) The control group consisted of 16 healthy women who referred to the Royan Institute for IVF/ICSI due to primary infertility. These women did not suffer from certain diseases and the cause of their infertility was mild to moderate male factor (Fig. 1).

All the participants in this study were at reproductive age (15–49 years). They did not take vitamin D supplements or Oral Contraceptive Pills (OCPs) for at least three months before stimulation, and were not on a special diet such as a vegetarian diet. Moreover, they were not taking certain medications like laxatives, steroids, cholesterol-lowering drugs, seizure-control drugs, rifampin, and orlistat. None of them had specific diseases such as liver disease, kidney disease, celiac disease, cystic fibrosis and Crohn's disease, or history of weight loss surgeries that reduce the size of the stomach and/or bypass part of the small intestine. Women with poor ovarian response according to Bologna criteria^31^, polycystic ovary syndrome according to Rotterdam criteria^32^, endometriosis, and body mass index (BMI) higher than 30 kg/m^2^ were not included in the study.

The eligible women were recruited in the study after obtaining written consent and answering a questionnaire, including demographic information, and questions regarding fertility, sun exposure, and physical activity. After evaluation of the pelvic ultrasound and hormonal profiles including Follicle-Stimulating Hormone (FSH) mIU/mL, Luteinizing Hormone (LH) mIU/mL and Anti-Mullerian Hormone (AMH) ng/mL, ovarian stimulation with Gonadotropin-Releasing Hormone (GnRH) antagonist protocol was started in both groups.

The control group and women with BC who were in the early follicular phase received a starting dosage of recombinant FSH on day 2–3 of the menstrual cycle for four or five consecutive days, but in the BC women who were in the late follicular/luteal phase, ovarian stimulation was done with a random start^33^. The initial dose was determined by the patient's age, BMI, AMH, and Antral Follicle Count (AFC). Serial folliculometry was performed by ultrasound, and subsequently, the gonadotropin dose was adjusted according to the ovarian response. The GnRH antagonist (Cetrorelix, Cetrotide® 0.25 mg, Serono, Germany) started at 12 mm follicle size and continued until the trigger day. When at least two follicles had reached 17–18 mm, ovulation was triggered with 0.2 mg of Triptorelin (GnRH agonist, Decapeptyl^®^, Ferring GmbH, Kiel, Germany) in BC women and 1 ml of Recombinant Human Chorionic Gonadotropin (r-hCG) (Ovitrelle^®^ 250 µg/0.5 ml, Merck, Germany) in the control group. After 34–36 h, oocyte retrieval was performed via transvaginal ultrasound-guided. In the women with BC, Letrozole 5 mg/day (Femati^®^, Atipharmed, Iran) was used from the beginning of ovarian stimulation and continued until oocyte retrieval.

Serum and FF samples were collected from all women on the day of oocyte retrieval and kept frozen at − 80 °C. At the end of sampling stage, serum and FF 25(OH) D levels were measured using the Electrochemiluminescence (ECL) Cobas^®^ Elecsys Vitamin D Total (Roche Diagnostics, Germany). In this study, serum 25-hydroxyvitamin D (25(OH) D) deficiency was considered less than 20 ng/ml; its insufficiency was 20–30 ng/ml and its replete level was more than 30 ng/ml^34,35^. FF 25(OH) D norm remains unknown. To obtain clear FF and prevent contamination by the flushing medium during the aspiration procedure or the FF of other follicles, the samples were aspirated only from the available lead follicle. The FF samples were centrifuged at 10,000 rpm for 10 min and the supernatants were stored at − 80 °C.

On the day of oocyte retrieval, the oocytes in each woman were evaluated by an embryologist for (a) the number of oocytes retrieved, (b) the number of Metaphase II (MII) oocytes, (c) oocyte maturity (number of MII oocytes/total number of oocytes × 100), and (d) quality of MII oocytes. Oocyte dysmorphisms were divided into cytoplasmic anomalies (e.g., presence of vacuoles, refractile bodies, increased granularity, and smooth endoplasmic reticulum aggregations) and extracytoplasmic anomalies (e.g., irregular shape or thickness of the zona pellucida, abnormal morphology of the first polar body, and perivitelline space)^29^. In the Royan Institute, the quality of MII oocytes is classified into four categories based on morphological criteria: Morph, Slightly dysmorph, Dysmorph and Highly dysmorph^36^.

### Statistical analysis

The collected data were analyzed using the Statistical Package for Social Sciences (SPSS) software (ver. 20). Qualitative and quantitative data were presented using frequency (percent) and mean (standard deviation). According to Farzadi et al., the difference in the mean levels of vitamin D between the pregnant and non-pregnant women was 4.2 units (11.5 ng/ml vs. 15.8 ng/ml i with a assumed standard deviation of equal 4.5) and type I and II errors of 0.05 and 0.20, The total number of estimated sample size was 36 (18 in each group)^26^. During the study, two participants in each group left the study and finally 16 people in each group entered the study. The data were summarized as mean ± SD or n (%). Normality testing was performed with the Shapiro–Wilk normality test. Independent t-test and Mann–Whitney’s U test were used to compare the means between the two independent quantitative variables and Chi-square test was used for qualitative variables. Correlation between the variables was calculated by using the Spearman/Pearson’s correlation coefficient. Kruskal–Wallis test was employed for comparison between all classes of one variable with different number of samples. P < 0.05 was considered statistically significant.

### Ethics approval and consent to participate

The study protocol was approved by the Research Ethics Committee of Tarbiat Modares University of Medical Sciences (Code: IR.MODARES.REC.1398.157). All procedures were in accordance with the ethical standards of the Regional Research Committee and with the Declaration of Helsinki 1964 and its later amendments. After explaining the research purposes, informed written consent and verbal assent were obtained from all participants. They were informed that their participation was voluntary, confidential and anonymous, and that they had the right to withdraw from the research at any time.

## Data Availability

The data sets used and analyzed for the current study are available upon reasonable request of the corresponding authors (Dr. Shahideh Jahanian shahideh.jahanian@modares.ac.ir and Dr. Firouzeh Ghaffari ghafaryf@yahoo.com).

## Abbreviations

BC: Breast cancer
VDR: Vitamin D receptor
FF: Follicular fluid
IVF: In vitro fertilization
ICSI: Intracytoplasmic sperm injection
BMI: Body mass index
FSH: Follicle-stimulating hormone
LH: Luteinizing hormone
AMH: Anti-mullerian hormone
GnRH: Gonadotropin-releasing hormone
HCG: Human chorionic gonadotropin
r-hCG: Recombinant human chorionic gonadotropin
25(OH) D: 25-Hydroxyvitamin D
ECL: Electrochemiluminescence
SPSS: Statistical package for social sciences
SD: Standard deviation
