# Author Correction: Evaluation of the relationship between vitamin D levels with oocyte quality in breast cancer women: a cross-sectional study

**DOI:** 10.1038/s41598-023-42122-0

**Published:** 2023-09-12

**Authors:** Mahshid Gharagozloo, Shahideh Jahanian Sadatmahalleh, Mehri Kalhor, Firouzeh Ghaffari, Fatemeh Hasani, Nadia Jahangiri, Malihe Nasiri, Ahmad Khosravi

**Affiliations:** 1 Department of Reproductive Health and Midwifery, Faculty of Medical Sciences, Tarbiat Modares University, Tehran, 14115-111 Iran; 2 Department of Midwifery, School of Nursing and Midwifery, Shahid Beheshti University of Medical Sciences, Tehran, Iran; 3 Department of Endocrinology and Female Infertility, Reproductive Biomedicine Research Center, Royan Institute for Reproductive Biomedicine, ACECR, Royan Allay, Eastern Hafez St., Banihashem Sq., Resalat Highway, Tehran, 1665659711 Iran; 4 Department of Embryology, Reproductive Biomedicine Research Center, Royan Institute for Reproductive Biomedicine, ACECR, Tehran, Iran; 5 Department of Basic Sciences, Faculty of Nursing and Midwifery, Shahid Beheshti University of Medical Sciences, Tehran, Iran; 6 Department of Epidemiology, School of Public Health, Ophthalmic Epidemiology Research Center, Shahroud University of Medical Sciences, Shahroud, Iran

Correction to: *Scientific Reports* 10.1038/s41598-023-39341-w, published online 26 July 2023

The original version of this Article contained an error in Affiliation 6, which was incorrectly given as ‘Department of Epidemiology, School of Public Health, Ophthalmic Epidemiology Research Center Shahroud, University of Medical Sciences, Tehran, Iran.’ The correct affiliation is listed below:

Department of Epidemiology, School of Public Health, Ophthalmic Epidemiology Research Center, Shahroud University of Medical Sciences, Shahroud, Iran.

The original Article has been corrected.

